# Food‐related salience processing in healthy subjects during word recognition: Fronto‐parietal network activation as revealed by independent component analysis

**DOI:** 10.1002/brb3.887

**Published:** 2017-12-18

**Authors:** Annette Safi, Christoph Nikendei, Valentin Terhoeven, Matthias Weisbrod, Anuradha Sharma

**Affiliations:** ^1^ Department of General Internal Medicine and Psychosomatics Centre for Psychosocial Medicine University Hospital Heidelberg Heidelberg Germany; ^2^ Research Group Neurocognition Department of General Psychiatry Centre for Psychosocial Medicine University Hospital Heidelberg Heidelberg Germany; ^3^ Department of Psychiatry and Psychotherapy SRH Hospital Karlsbad‐Langensteinbach Karlsbad Germany

**Keywords:** dipole source analysis, electroencephalography, food salience, fronto‐parietal network, independent component analysis, recognition memory

## Abstract

**Background:**

The study aimed to isolate and localize mutually independent cognitive processes evoked during a word recognition task involving food‐related and food‐neutral words using independent component analysis (ICA) for continuously recorded EEG data. Recognition memory (old/new effect) involves cognitive subcomponents—familiarity and recollection—which may be temporally and spatially dissociated in the brain. Food words may evoke additional attentional salience which may interact with the old/new effect.

**Methods:**

Sixteen satiated female participants undertook a word recognition task consisting of an encoding phase (learning of presented words, 40 food‐related and 40 food neutral) and a test phase (recognition of previously learned words and new words). Simultaneously recorded 64‐channel EEG data were decomposed into mutually independent components using the Infomax algorithm in EEGLAB. The components were localized using single dipole fitting using a four‐shell BESA head model. The resulting (nonartefactual) components with <15% residual variance were clustered across subjects using the kmeans algorithm resulting in five meaningful clusters localized to fronto‐parietal regions. Repeated‐measures anova was employed to test main effects (old/new and food relevance) and their interaction on cluster time courses.

**Results:**

Early task‐relevant old/new effects were localized to the medial frontal gyrus (MFG) and later old/new effects to the right parietal regions (precuneus). Food‐related (nontask‐relevant) salience effects were localized to bilateral parietal regions (left precuneus and right postcentral gyrus). Food‐related salience interacted with task relevance, the old/new effect in MFG being significant only for food‐neutral words highlighting central the role of MFG as the converging site of endogenous and exogenous salience inputs.

**Conclusion:**

Our results indicate ICA to be a valid technique to decompose complex neurophysiological signals involving multiple cognitive processes and implicate the fronto‐parietal network as an important attentional network for processing salience and task demands.

## 1 INTRODUCTION

Recognition of words is known to engage semantic as well as memory networks in the brain (Hauk, Davis, Ford, Pulvermuller, & Marslen‐Wilson, 2006; Nelson, Kitto, Galea, McEvoy, & Bruza, 2013). Its disturbance has been implicated in several psychiatric disorders such as schizophrenia (Kayser et al., 2010), depression (Suslow, 2009; van Tol et al., 2012), and eating disorders (Nikendei et al., 2008, 2012; Terhoeven et al., 2016). Recognition memory has been divided into two broad cognitive subcomponents—familiarity (a feeling of knowing a presented item without contextual details) and recollection (retrieval of additional contextual information of a previously studied item); (Jacoby, 1991; Mandler, 1980; Yonelinas, Otten, Shaw, & Rugg, 2005). Familiarity is known to operate early on in the process of recognition and has been linked to an early event‐related potential (ERP) over the frontal scalp sites between 300 and 500 ms poststimulus onset (Rugg & Curran, 2007). Recollection on the other hand is reported to be active later on and has been linked to an ERP component between 500 and 800 ms poststimulus onset over the posterior scalp sites (Rugg & Curran, 2007). In this context, the old/new effect can be defined as a divergent ERP wave, reflecting the awareness that an object has previously been perceived (Mecklinger, 2000).

Functional brain imaging literature on recognition memory has reported different brain regions to be active during familiarity and recollection processes. Anterior parahippocampal gyrus, lateral prefrontal cortex, superior parietal cortex, insula, and cerebellum regions have been reported to be involved in familiarity (Aggleton & Brown, 2006; Skinner & Fernandes, 2007; Yonelinas et al., 2005). Recollection has been linked to posterior parahippocampal gyrus, hippocampus, anterior medial prefrontal cortex, postcentral gyrus, lateral parietal cortex, and inferior temporal gyrus regions (Diana, Yonelinas, & Ranganath, 2007; Spaniol et al., 2009; Yonelinas et al., 2005). There are also regions involved in both familiarity and recollection, for example, the left precuneus (Dorfel, Werner, Schaefer, von Kummer, & Karl, 2009). So far, few studies have tried to map the familiarity and recollection‐related ERPs onto brain regions observed to be activated in fMRI during these processes (Bergstrom, Henson, Taylor, & Simons, 2013; Hoppstadter, Baeuchl, Diener, Flor, & Meyer, 2015). Moreover, the limited time resolution of the fMRI‐BOLD signal allows only indirect connection to be drawn to the recognition‐related ERPs.

The intake of food is crucial for our survival. Therefore, food stimuli are known to evoke attention and activate brain networks processing highly salient objects and words. Food stimuli compared to nonfood stimuli (pictures and words) have been found to activate different brain regions, namely occipital, limbic, paralimbic, and prefrontal areas (Appelhans, 2009; Kringelbach, 2004). Food‐related pictures are also reported to evoke different behavioral and neural network reactions depending on variable states of hunger (Goldstone et al., 2009; Piqueras‐Fiszman, Kraus, & Spence, 2014; Stockburger, Schmalzle, Flaisch, Bublatzky, & Schupp, 2009), body weight (Karhunen et al., 2000), restrained versus unrestrained eating behavior (Veenstra & de Jong, 2010), and diagnosis of eating disorders such as binge eating disorder and anorexia nervosa (Godier, Scaife, Braeutigam, & Park, 2016; Karhunen et al., 2000; Nikendei et al., 2012). Most studies reviewed above investigating neural correlates of food‐related stimuli are fMRI studies that have investigated responses to food‐related pictures (Garcia‐Garcia et al., 2013; Tataranni & DelParigi, 2003). There is much less literature on processing of food‐related words which has mainly investigated ERPs in response to food‐related words (Leland & Pineda, 2006; Nijs, Franken, & Muris, 2010). EEG can complement fMRI research on food stimuli processing due to enhanced temporal resolution, but lacks the appropriate spatial resolution provided by fMRI.

This study therefore aimed to apply a novel analysis strategy independent component analysis (ICA) to decompose continuously recorded EEG data into mutually independent clusters localized to respective brain areas to obtain a higher spatial resolution for EEG signal. ICA is a method that allows the decomposition of complex neurophysiological signals into mutually independent components which include both artefactual and neurally generated signals (Delorme & Makeig, 2004). This method, however, has been so far mostly applied for the rejection of artefactual data in EEG studies and its utility for decomposing complex and superimposed cognitive processes is scarcely investigated.

Only two studies have employed ICA in word‐processing tasks (Mehta, Jerger, Jerger, & Martin, 2009; Summerfield & Mangels, 2005). While Summerfield and Mangels (2005) applied ICA to time‐frequency and coherence data into independent components. However, the number of components was restricted a priori to a maximum of four components limiting the spatial resolution of the decomposition and restricting the localization analysis only to major sources/networks. Mehta et al. (2009) used ICA on group data containing only 30 channel EEG data and based the component extraction on time‐course analysis and not on localization of components. In this study, we employed a localization‐led dipole clustering approach post‐ICA to cluster mutually independent components from continuous EEG data into corresponding regional brain activations in a sample of 16 healthy subjects undertaking a word recognition task. Specifically, we aimed to localize activities of early (predominantly) familiarity‐related and late (predominantly) recollection‐related effects. As the task involved the recognition of both food‐related and food‐neutral words, we further investigated the interaction of implicit food‐related salience with the task‐relevant familiarity and recollection‐related processes.

The study involves a retrospective analysis of data from a study, for which traditional ERP analysis has been previously published (Nikendei et al., 2012). This data provides a good set for the exploratory validation of ICA as it involved two different cognitive processes (recognition memory and food relevance), which would evoke activity in different brain areas/networks and allow the examination of the utility of ICA for decomposing mixed‐up cognitive processes superimposed on different brain areas.

This is the first study to employ ICA to elucidate such interaction based on regional localizations of components while retaining the high time resolution offered by EEG and confirms the validity of this method for decomposing complex neurophysiological signals involving several cognitive processes. The study will provide important insights into localization of neural processes related to food words as most previous investigations of food‐related words (Leland & Pineda, 2006; Nijs et al., 2010) have employed traditional ERP analysis focusing on the time course of word processing rather than spatial aspects. The study will moreover throw light on the interaction of two complex processes (recognition memory and food‐related processing), disturbances in both of which are implicated in eating disorders such as anorexia nervosa (Hermans, Pieters, & Eelen, 1998; Nikendei et al., 2011; Pietrowsky, Krug, Fehm, & Born, 2002). The results from the study highlight spatial and temporal interaction in the brain of food relevance with recognition memory and have implications for elucidating neural mechanisms resulting in psychopathological cognitive biases observed in eating disorders.

## 2 MATERIALS AND METHODS

### 2.1 Participants

Sixteen female satiated participants (mean age = 22.8 years) with food intake before the experiment took part in this study. The data analyzed in this study were collected as part of a larger study conducted between 2004 and 2006 investigating recognition memory in satiated and fasting anorexia nervosa patients and healthy controls (Nikendei et al., 2008, 2012). In this study, we analyzed data from healthy satiated participants undertaking a word recognition paradigm, where participants were required to identify words (food‐related and food‐neutral) previously seen during the encoding phase with a button press. Inclusion criteria were age between 18 and 35 years, normal body weight (Body Mass Index [BMI] between 18.5 and 24.9 kg/m^2^), right‐handedness, normal or corrected‐to‐normal vision, and native German language. Exclusion criteria were a life‐threatening medical condition, a medical history of psychosis or craniocerebral injury and psychopharmacological medication. In addition, all participants underwent semistructured interviews to evaluate lifetime diagnoses of eating disorders and severe psychiatric disease (Fichter, Herpertz, Quadflieg, & Herpertz‐Dahlmann, 1998; Wittchen, Zaudig, & Fydrich, 1997), as these diagnoses also led to exclusion. Participants were required to avoid drinking caffeinated beverages for 1 hr and alcohol for 24 hr before the experiment.

The study was approved by the local ethical committee (no. 281/20‐03) of the medical faculty of University of Heidelberg, and all participants provided written informed consent according to the Declaration of Helsinki (Fifth Revision, 2000).

### 2.2 Word stimuli

Word stimuli consisted of 80 food‐related and 80 nonfood‐related nouns. In order to find comparable word samples, for each food‐related word, a neutral nonfood‐related word had been selected, matched for the number of letters and number of syllables and for frequency of usage in the German language. Information concerning frequency of usage was acquired in cooperation with the Institute of German Language (Institut für Deutsche Sprache; IDS) in Mannheim, Germany. Two sets, each consisting of 40 food‐related word stimuli and 40 corresponding neutral word stimuli, were used for the encoding phase and for the subsequent recognition test. The two sets did not vary with respect to the number of syllables, number of letters and frequency of usage and approximate caloric content. The latter was estimated by a dietician. Emotional valence of food‐related and neutral word stimuli was not significantly different, when assessed in a pretest in a sample of six healthy subjects using Self‐Assessment Manikins (SAM) as a nonverbal pictorial assessment technique for person's affective reaction to each word stimulus (Bradley & Lang, 1994; Nikendei, Schild, Voelkl, Herzog, & Zipfel, 2005).

### 2.3 Procedure

There were two phases consisting of encoding phase and a subsequent recognition test, following immediately after one another. Words were displayed in uppercase 46‐point Times New Roman font. Before each encoding phase of the experiment, participants received written instructions displayed on the monitor. Practice trials with neutral words were conducted to familiarize participants with the trial sequence.

During the encoding phase, 80 target words, consisting of 40 food‐related stimuli and 40 neutral stimuli, and 80 distractors, consisting of geometric forms, were presented in a random sequence. The geometric forms consisted of medium‐sized black and white simple shapes, such as squares, circles, and triangles, and were employed in order to increase the level of attention during the encoding phase. Participants were asked to respond to the presentation of geometric forms by pressing the “yes” response button held in the dominant right hand as quickly and as accurately as possible. Participants were also asked to remember target words as accurately as possible. In contrast to the presentation of geometric forms, words therefore did not require a response. Each of the 160 trials lasted for 4 s. Each trial involved the presentation of a fixation cross in the center of the screen for a duration of 800 ms followed by a 200‐ms presentation of the target or distractor. A response was possible within the next 2 s. The intertrial interval was set at 1 s. Thus, the encoding phase lasted for 11 min.

During the recognition test, 160 words were displayed in a random sequence, consisting of two sets of 40 food‐related words and two sets of 40 neutral words. One set from each category had been presented in the encoding phase, whereas the other two sets were new to the participants. Participants were asked to indicate whether the presented word had been previously presented in the experiment as quickly and as accurately as possible. Participants held one response button in their left hand and one response button in their right hand. “Yes” or “no” responses were made by pressing the button in either their left or their right hand, respectively. Each of the 160 trials lasted for 4 s. Each trial involved the presentation of a fixation cross in the center of the screen for 800 ms followed by a 200‐ms presentation of a word stimulus. A response was possible within the next 2 s. The intertrial interval was set at 1 s. The recognition test lasted for 11 min.

### 2.4 EEG recordings and data preprocessing

Participants were seated 1 m in front of the video graphics array monitor in an electrically shielded, sound‐attenuated, and dimly lit chamber. EEG was continuously recorded with a direct current amplifier (Quickamp; BrianProcducts, Munich, Germany) with a sampling rate of 1000 Hz and a resolution of 0.1 μV using a 64‐channel electrode cap (Falk Minow Services, Herrsching, Germany) with sintered Ag/AgCl electrodes placed according to an extended international 10–20 system during the recognition test. Horizontal and vertical electrooculogram (EOG) was recorded by electrodes 1 cm next to the outer canthi (horizontal EOG) and above and below the left eye (vertical EOG). All impedances were kept <5 kΩ. Data were recorded with 200 Hz (antialiasing) filter and a notch filter at 50 Hz with Cz as the reference electrode.

EEG data were processed using the software EEGLAB (Delorme & Makeig, 2004) based in MATLAB (The Mathworks, Inc. Massachusetts, USA). Continuous EEG data were filtered using a 1 Hz low cut‐off basic FIR filter as implemented in EEGLAB to remove low‐frequency noise and slow drifts. Segments containing large irregular movement‐related artifacts and noise were removed by visual screening, and data were rereferenced to average reference as suggested by the current guidelines of EEGLAB (Delorme & Makeig, 2004) and also implemented by the previous studies (e.g., Summerfield & Mangels, 2005).

### 2.5 Independent component and dipole source analysis

To decompose the filtered and artifacts‐cleaned EEG data into temporally, functionally, and spatially independent source signals, we applied independent component analysis (ICA) using the extended Infomax runica algorithm as implemented in EEGLAB. This led us to 64 independent components (ICs) each with mutually independent time courses and scalp topographies for each participant. ICs depicting physiological artefacts (eye movements and eye blinks, muscle activity, cardiac pulse artifacts) as well as artefacts consisting of line noise and spatially irregular components of unknown origin were removed via visual inspection. Dipole source analysis was performed using the DIPFIT function in EEGLAB, and one single dipole was fitted for every IC using a four‐shell BESA head model as the brain structural template. ICs having dipole residual variance of more than 15% as well as with dipoles lying outside brain regions were excluded from further analysis. The continuous preprocessed EEG data were segmented around the four types of word triggers (food‐related words presented in encoding phase, neutral words presented in encoding phase, food‐related words not previously presented, neutral word not previously presented) yielding epochs of −400 and 2,200 ms centered around the stimulus appearance. Only segments including correct hits were included in further analysis. ICA weights were applied to segmented EEG data and segments were averaged and baseline corrected with −400 to 0 ms as the baseline interval to generate ERPs for ICs for every subject and condition.

### 2.6 Component clustering across subjects

To identify ICs across subjects corresponding to similar brain sources, components from all subjects were preclustered using principle component analysis (PCA) in EEGLAB based on scalp topographies and dipole locations. The preclustered components were then clustered using “kmeans” clustering algorithm (as implemented in EEGLAB) with number of clusters preset to 15 to start with. Based on the distribution of obtained clusters, the number of clusters was consecutively brought down to seven to obtain clusters containing corresponding components from a high proportion of subjects. One resulting cluster containing components with extremely scattered dipole locations was excluded and not examined further. One more cluster contained components from few subjects (*n* = 12) and was also excluded from further analysis resulting in a final number of five clusters containing components from every subject and a cluster centroid location for every cluster. If one participant contributed more than one IC to the cluster, the poorer matching ICs were removed from the cluster via visual inspection based on closeness to cluster centroid until only one IC per participant remained in the cluster. If one of participants did not contribute any IC to the cluster, we visually examined the dipole locations and scalp topographies of individual components to find a matching IC (based on dipole location and closeness to the corresponding cluster centroid) of the previously missing participant and reassigned it to the relevant cluster. Given the small sample size of the study, we ensured a priori that only clusters that had components from all subjects were included in statistical analysis to avoid confounding of the statistical analysis from the lack of power for incomplete clusters. Cluster centroid locations were assigned to gray matter brain regions using the software Talairach Client (http://www.talairach.org/client.html) which reports Talairach labels for user‐defined coordinates for nearest gray matter locations.

### 2.7 Statistical analysis

Based on systematic literature review on recognition memory and the visual inspection of the IC cluster ERP time courses, we analyzed two‐time windows for the elucidation of early (300–500 ms) and late (500–700 ms) old/new effects in the obtained clusters. These time windows have been previously reported for in early (mainly familiarity) and later (mainly recollection) influences of previously seen stimuli (Hoppstadter et al., 2015; Rugg & Curran, 2007; Vilberg & Rugg, 2008). We furthermore examined the effect of food relevance on the cluster ERPs in these time windows to examine the interaction of food‐related salience with old/new effects. Repeated‐measures anovas were performed using the SPSS software (PSS Inc., Chicago, IL) separately for the early and late time intervals with mean ERP voltages during the relevant time interval as the dependent variable and old/new (words previously presented during encoding phase vs. new words) and food relevance (food‐related words vs. food‐neutral words) as repeated‐measure factors. Main and interaction effects that reached statistical or trend‐level significance are reported along with effect sizes (partial eta^2^ small 0.01, medium 0.06, large 0.14). Post hoc comparisons were performed using Fisher's LSD test when interactions reached significance or trend‐level significance. The analysis was conducted separately for every cluster to localize the obtained statistical effects to the relevant brain regions as indicated by cluster dipole centroid locations.

## 3 RESULTS

### 3.1 Participants and cluster properties

Sixteen female satiated and normal weight women participated in this study. The mean BMI was 20.9 kg/m^2^ (1.7) and the mean age was 23.6 (5.2).

**Figure 1:**
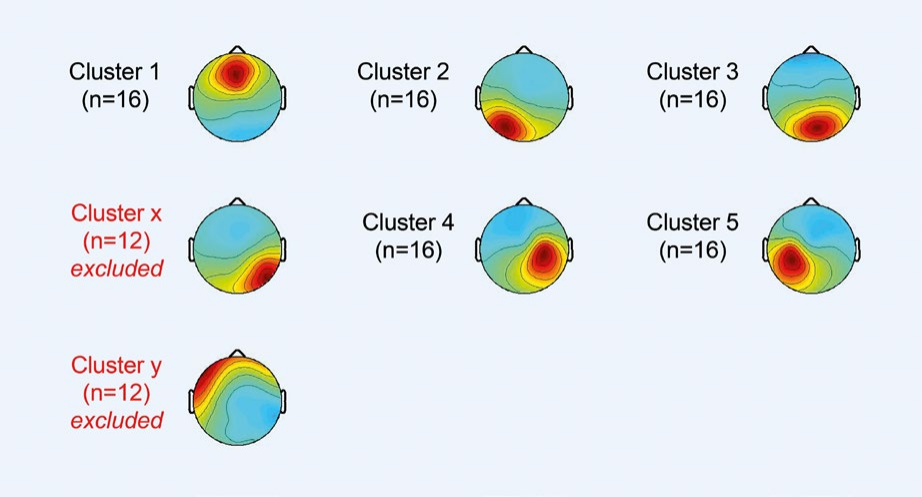
Average scalp maps for cluster centroids of all participants (*n* = 16) are shown for each obtained cluster

**Figure 2:**
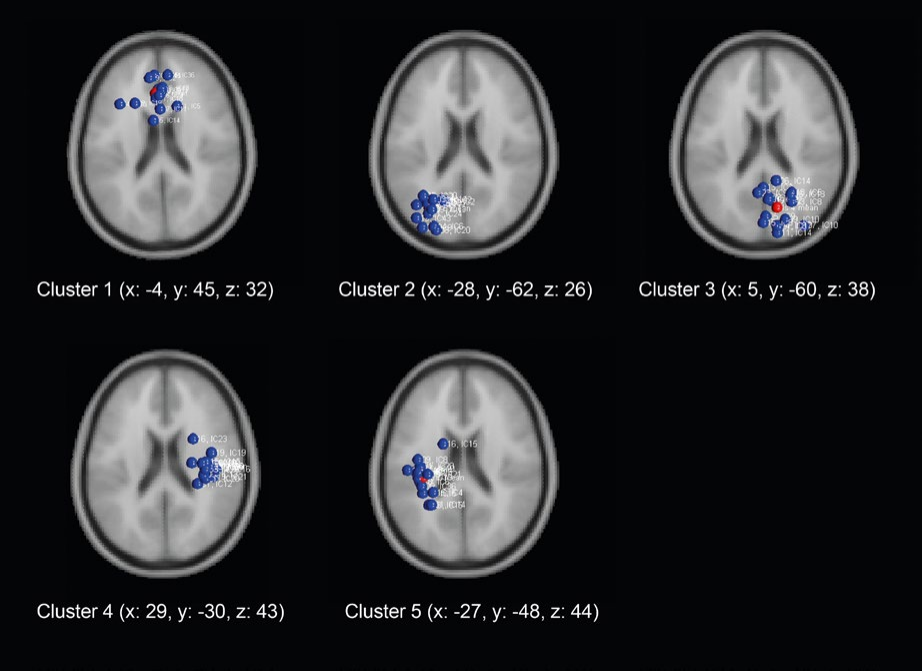
Localizations of the independent component dipoles from individual subjects (*n* = 16) within every cluster are shown along with the cluster centroid locations (Talairach coordinates)

Scalp topographies for the cluster centroids are depicted in Figure 1 and obtained clusters containing individual IC dipoles from all subjects are shown in Figure 2. Talairach coordinates and nearest gray matter location for cluster centroids are reported in Table 1.

**Table 1:** Localizations of cluster centroids are shown in Talairach coordinates along with the corresponding nearest gray matter locations and Brodmann areas

Cluster	Talairach coordinates	Gray matter location and nearest/adjacent Brodmann areas (BA)
Cluster 1	*x*: −4, *y*: 45, *z*: 32	Medial frontal gyrus, BA9
Cluster 2	*x*: −28, *y*: −62, *z*: 26	Left temporal Lobe, middle temporal gyrus, BA 39/40
Cluster 3	*x*: 5, *y*: −60, *z*: 38	Right parietal lobe, precuneus, BA 7
Cluster 4	*x*: 29, *y*: −30, *z*: 43	Right parietal lobe, postcentral gyrus, BA 3
Cluster 5	*x*: −27, *y*: −48*, z*: 44	Left parietal lobe, precuneus, BA7

### 3.2 Early and late old/new and food‐related effects

Repeated‐measures anova main effects and interactions for the early (300–500 ms) and late (500–700 ms) time windows are reported in Table 2.

**Table 2:** Repeated‐measures anovas testing for main effects (old/new and food relevance) and interaction effects on cluster time courses in the early (300–500 ms) and late (500–700 ms) time windows are given in the table. Effect sizes (partial *eta* ^2^): small 0.01 – medium 0.06 – large 0.14

Cluster	Time window	anova main effects	anova interaction effects (Old/new × Food relevance)
Cluster 1	Early	Old/new (*F*(1,15) = 3.36, *p* = .087, partial‐eta^2^ = 0.18)^b^Food relevance (*F*(1,15) = 0.49, *p* = .493, partial‐eta^2^ = 0.03)	*F*(1,15) = 3.40, *p* = .085, partial‐eta^2^ = 0.18^b^
	Late	Old/new (*F*(1,15) = 0.00, *p* = .993, partial‐eta^2^ = 0.00)Food relevance (*F*(1,15) = 1,67, *p* = .216, partial‐eta^2^ = 0.10)	*F*(1,15) = 2.44, *p* = .139, partial‐eta^2^ = 0.14
Cluster 2	Early	Old/new (*F*(1,15) = 2.53, *p* = .133, partial‐eta^2^ = 0.14)Food relevance (*F*(1,15) = 1.79, *p* = .201, partial‐eta^2^ = 0.11)	*F*(1,15) = 0.30, *p* = .591, partial‐eta^2^ = 0.02
	Late	Old/new (*F*(1,15) = 1.36, *p* = .261, partial‐eta^2^ = 0.08)Food relevance (*F*(1,15) = 2.60, *p* = .128, partial‐eta^2^ = 0.15)	*F*(1,15) = 0.21, *p* = .654, partial‐eta^2^ = 0.01
Cluster 3	Early	Old/new (*F*(1,15) = 1.14, *p* = .303, partial‐eta^2^ = 0.07)Food relevance (*F*(1,15) = 0.31, *p* = .584, partial‐eta^2^ = 0.02)	*F*(1,15) = 0.12, *p* = .736, partial‐eta^2^ = 0.01
	Late	Old/new (*F*(1,15) = 3.10, *p* = .099, partial‐eta^2^ = 0.17)^b^Food relevance (*F*(1,15) = 1.78, *p* = .201, partial‐eta^2^ = 0.11)	*F*(1,15) = 1.93, *p* = .185, partial‐eta^2^ = 0.11
Cluster 4	Early	Old/new (*F*(1,15) = 1.07, *p* = .318, partial‐eta^2^ = 0.07)Food relevance (*F*(1,15) = 0.00, *p* = .99, partial‐eta^2^ = 0.00)	*F*(1,15) = 0.07, *p* = .799, partial‐eta^2^ = 0.00
	Late	Old/new (*F*(1,15) = 1.18, *p* = .294, partial‐eta^2^ = 0.07)Food relevance (*F*(1,15) = 3.21, *p* = .093, partial‐eta^2^ = 0.18)^b^	*F*(1,15) = 0.20, *p* = .659, partial‐eta^2^ = 0.01
Cluster 5	Early	Old/new (*F*(1,15) = 0.61, *p* = .449, partial‐eta^2^ = 0.04)Food relevance (*F*(1,15) = 0.01, *p* = .917, partial‐eta^2^ = 0.00)	*F*(1,15) = 0.30, *p* = .592, partial‐eta^2^ = 0.02
	Late	Old/new (*F*(1,15) = 0.02, *p* = .884, partial‐eta^2^ = 0.00)Food relevance (*F*(1,15) = 4.75, *p* = .046, partial‐eta^2^ = 0.24)^a^	*F*(1,15) = 0.30, *p* = .591, partial‐eta^2^ = 0.02

a Effect was statistical significant at alpha = 0.05.

b Effect exhibited trend‐level significance (.05 < *p* < .1).

For the frontal (medial frontal gyrus), Cluster 1 trend‐level old/new effect (previously seen words evoking a larger positive potential) with large effect size ((*F*(1,15) = 3.36, *p* = .087, partial‐eta^2 ^= 0.18) as well as a trend‐level old/new x food relevance interaction with large effect size (*F*(1,15) = 3.40, *p* = .085, partial‐eta^2 ^= 0.18) were found for the early (300–500 ms) time window. Fisher's LSD post hoc test revealed a significant difference between old and new conditions only for the food‐neutral condition (*p* = .02). For all other clusters, none of the main effects or interactions reached significance for the early (300–500 ms) time window.

For the later time window (500–700 ms), right parietal (precuneus) Cluster 3 showed trend‐level old/new effect (previously seen words evoking a larger positive potential) with a large effect size (*F*(1,15) = 3.10, *p* = .099, partial‐eta^2 ^= 0.17). Right parietal (postcentral gyrus) Cluster 4 showed trend‐level *F*(1,15) = 3.21, *p* = .093, partial‐eta^2 ^= 0.18) and left parietal (precuneus) Cluster 5 showed a significant main effect of food relevance (food‐related words evoking a larger positive potential) (*F*(1,15) = 4.75, *p* = .046, partial‐eta^2 ^= 0.24), both with large effect sizes. None of the interactions reached (trend‐level/) significance for these clusters.

**Figure 3:**
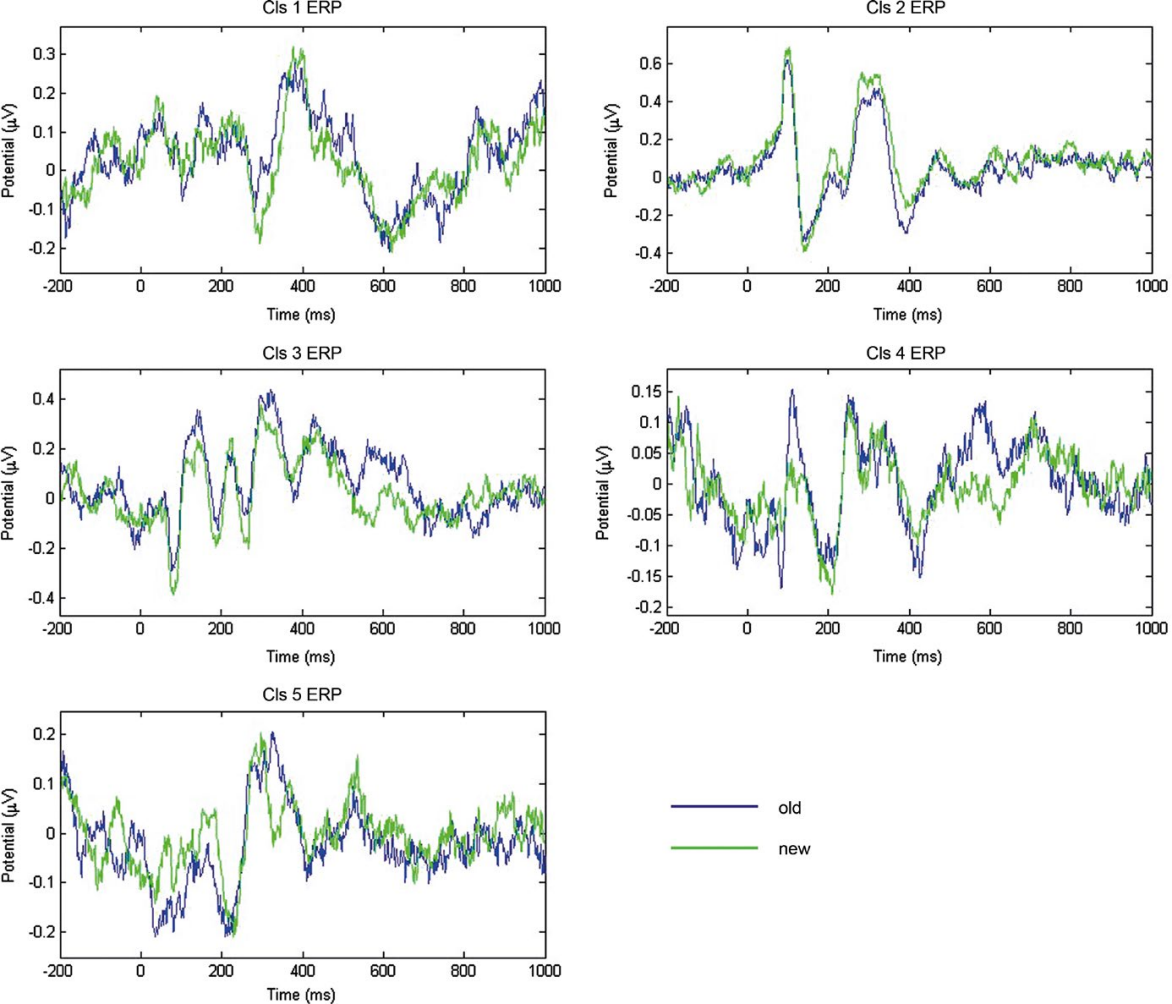
Figure depicts cluster ERPs (*n* = 16) of previously seen versus new words time‐locked to the onset of the word stimulus

**Figure 4:**
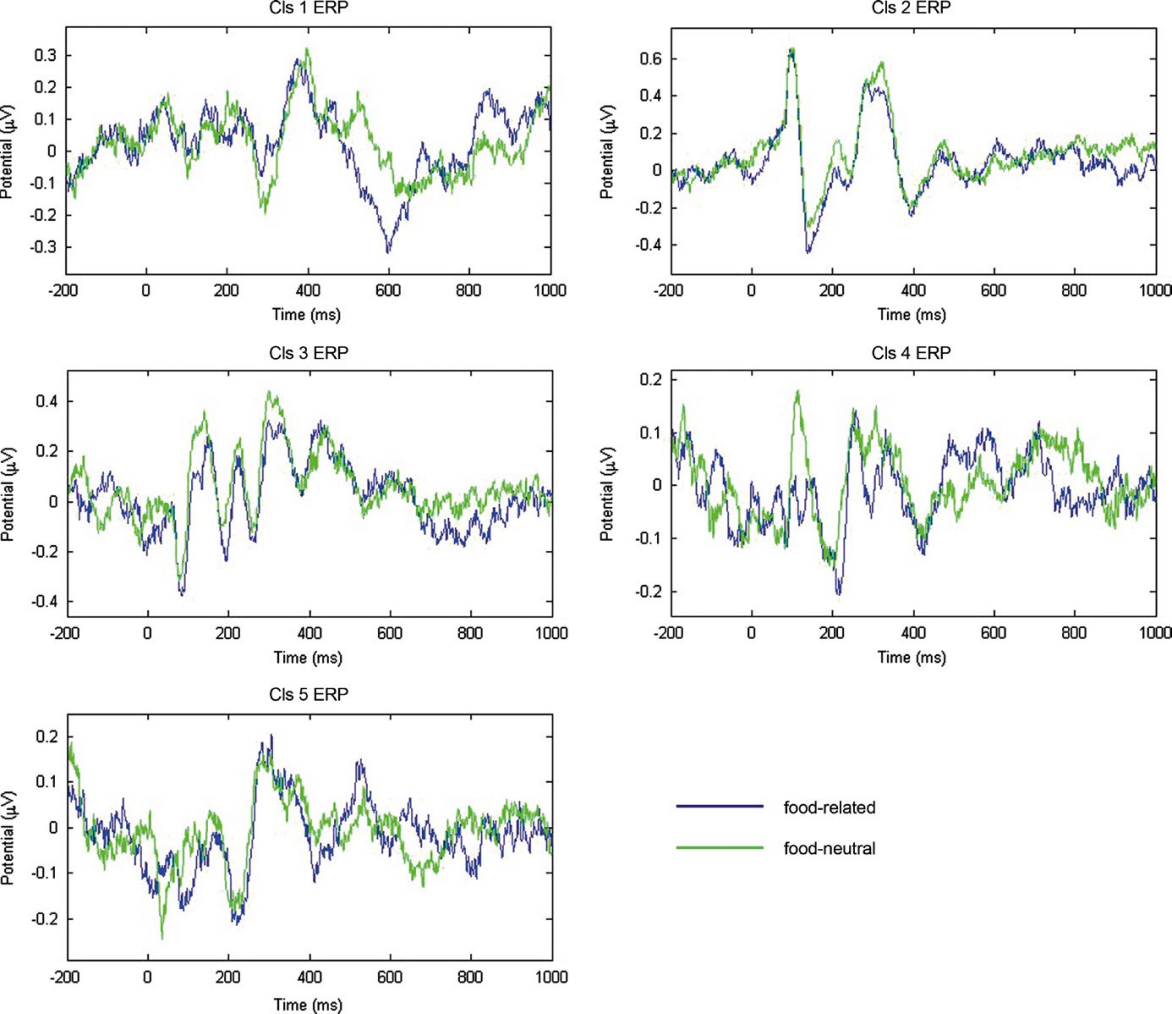
Figure depicts cluster ERPs (*n* = 16) of food‐related versus food‐neutral words time‐locked to the onset of the word stimulus

ERPs for every cluster for correct recognition trials are shown for the previously seen versus new word trials in Figure 3 and for food‐related versus food‐neutral word trials in Figure 4.

## 4 DISCUSSION

This is one of the few studies validating the applicability of ICA to continuously collected EEG data and utility and relevance of obtained component clusters for time‐course analysis and source‐localization of averaged cluster ERPs. The results show interesting patterns of task relevance and (likely) familiarity in early (300–500 ms) time window localized to the medial frontal gyrus. Later (likely) recollection effects (500–700 ms) on the other hand were localized to right parietal regions. Moreover, food‐related (nontask‐relevant) salience effects were localized to right and left parietal regions in the later time window (500–700 ms).

Our results fit well with the previous ERP literature on recognition and memory, which show earlier frontal positivity, most likely connected with familiarity of previously seen stimuli and later positive potentials over posterior scalp sites, consistently linked to deeper encoding and recognition of previously seen words (Duzel et al., 2003; Maratos, Allan, & Rugg, 2000; Schloerscheidt & Rugg, 2004). These results likewise fit well with fMRI studies localizing effects of task relevance and familiarity to (medial) frontal regions (Herzmann, Jin, Cordes, & Curran, 2012; Hoppstadter et al., 2015) and with recollection employing a further host of parietal brain structures (Herzmann et al., 2012; Yonelinas et al., 2005) including the right precuneus (Dorfel et al., 2009) as also indexed in the our results.

Our results further revealed an interaction between food relevance and old/new effect in the medial frontal gyrus, with a significantly pronounced old/new effect for food‐neutral words. This complex interaction could indicate an interference from implicit food‐related reward/salience in execution of task‐relevant cognitive processing (recognition of previously seen words), given the central role of medial frontal gyrus in the reward circuit (Haber & Knutson, 2010; Kaufmann et al., 2013). The medial fontal gyrus has been moreover postulated to be a converging site for the dorsal and ventral attentional networks, serving as a circuit‐breaker to interrupt ongoing endogenous attentional processes in the dorsal network and reorient attention to an exogenous stimulus (Japee, Holiday, Satyshur, Mukai, & Ungerleider, 2015) and playing a crucial role in overriding prepotent patterns and execution of task‐relevant responses (Aron, Robbins, & Poldrack, 2004). It is also reported to be activated when important task‐relevant cues are detected independent of the related response (Hampshire, Chamberlain, Monti, Duncan, & Owen, 2010). The significant old/new effects obtained for medial frontal gyrus cluster in our results only for food‐neutral conditions could indicate a resource conflict imposed by food‐related words impeding the successful execution of task‐relevant old/new rule‐set, which would be consistent with the role of medial frontal gyrus as the interacting site of different input streams (e.g., Talati & Hirsch, 2005) and task‐related resource allocation (Koshino et al., 2011). An interference of food salience in the execution of task‐relevant responses could provide an interesting mechanistic model for eating disorders such as anorexia nervosa where a heightened neural response to food stimuli has been reported in the reward circuit (e.g., Cowdrey, Park, Harmer, & McCabe, 2011). Consistent with the results of the current study, where reduced recognition memory effects were observed for the food‐related condition, abnormal neural responses in the reward/salience network (especially to food‐related stimuli) could provide pathological interference with several cognitive processes (e.g., attention, executive function) dependent on the fronto‐parietal network leading to cognitive impairment observed in eating disorders (e.g., reviewed in Zakzanis, Campbell, & Polsinelli, 2010).

In addition to the early interaction of food‐related salience with the old/new effect in the frontal cluster, food‐related effects (increased positivity) were localized in the later time window to the right and left parietal regions, specifically the right postcentral gyrus and the left precuneus. These regions are consistent with the previous studies which have reported these cortical areas to be involved in food‐related stimulus processing (as reviewed in Asmaro & Liotti, 2014; Evero, Hackett, Clark, Phelan, & Hagobian, 2012). There is hardly any literature on specific brain localization of processing of food‐related words, most previous studies focusing on temporal aspects of processing (Leland & Pineda, 2006; Nijs et al., 2010) and therefore, the study forms a significant contribution to the body of literature regarding the localization of food‐related effects, especially for word stimuli. Postcentral gyrus has been additionally implicated in target‐salience processing during the oddball‐task (Harsay, Spaan, Wijnen, & Ridderinkhof, 2012) and greater activation of this area in response to food‐related words in our results could indicate the automatic salience effects of food words operating during the task. These areas also form an important part of the fronto‐parietal attention network which integrates bottom‐up and top‐down information processing with posterior parietal areas strongly engaged in forming an attention priority map of external stimulus based on integration of sensory feature‐based as well as top‐down attentional effects (e.g., reviewed by Ptak, 2012). Higher reward and appetitive salience loading of food‐related words could explain heightened activation due to food‐related words compared to nonfood words in posterior parietal areas of the network. Taken together, these results highlight the strong attention evoking effects of food‐related stimulus which are perceived as salient even when this salience is irrelevant to the task and when the brain is simultaneously engaged in other performance‐relevant aspects of the task. The results furthermore indicate that the salience effects generated from perception of food‐related stimuli are strong enough to interfere with task‐relevant rule sets as shown by the pattern of interactions for the medial frontal cluster.

Limitations of the study were broad single dipole localizations of the clusters, the precise locations of which may vary according to individual head anatomy. Moreover, the small sample size leads to lower statistical power and only very strong effects were statistically visible as trends. Some smaller effects therefore may not have emerged at all during the analysis. The small sample size notwithstanding, the large effect sizes obtained for all trend‐level effects indicate statistically relevant results, which should be validated in future studies employing larger sample strength and a combination of neuroimaging techniques.

## 5 CONCLUSION

We were able to decompose the time course as well as broad localization of two important cognitive processes (word recognition and food salience) during one single task using a novel signal processing technique, ICA, the applicability of which to continuous EEG data is still not well‐established. Our results indicate early task‐related old/new effect localized to medial frontal region and later old/new effects as well as food‐related salience localized to posterior parietal regions. Interaction of food relevance with early old/new effect implicates frontal medial region in processing task‐related and salience demand conflicts as well as strong attentional salience evoked by food‐related words interfering with task demands. The strong overlap of localization and ERP time courses with previous neuroimaging literature implicate ICA to be a valid technique to decompose important cognitive processes reflected in EEG activity which may be otherwise mixed‐up in scalp channel data, enabling time‐course analysis (not possible with fine precision for other neuroimaging techniques) for mutually independent brain activities and their localization to corresponding brain regions.

## References

[brb3887-bib-0001] Aggleton, J. P. , & Brown, M. W. (2006). Interleaving brain systems for episodic and recognition memory. Trends in Cognitive Sciences, 10, 455–463. https://doi.org/10.1016/j.tics.2006.08.003 16935547 10.1016/j.tics.2006.08.003

[brb3887-bib-0002] Appelhans, B. M. (2009). Neurobehavioral inhibition of reward‐driven feeding: Implications for dieting and obesity. Obesity (Silver Spring), 17, 640–647. https://doi.org/10.1038/oby.2008.638 19165160 10.1038/oby.2008.638

[brb3887-bib-0003] Aron, A. R. , Robbins, T. W. , & Poldrack, R. A. (2004). Inhibition and the right inferior frontal cortex. Trends in Cognitive Sciences, 8, 170–177. https://doi.org/10.1016/j.tics.2004.02.010 15050513 10.1016/j.tics.2004.02.010

[brb3887-bib-0004] Asmaro, D. , & Liotti, M. (2014). High‐caloric and chocolate stimuli processing in healthy humans: An integration of functional imaging and electrophysiological findings. Nutrients, 6, 319–341. https://doi.org/10.3390/nu6010319 24434747 10.3390/nu6010319 PMC3916864

[brb3887-bib-0005] Bergstrom, Z. M. , Henson, R. N. , Taylor, J. R. , & Simons, J. S. (2013). Multimodal imaging reveals the spatiotemporal dynamics of recollection. NeuroImage, 68, 141–153. https://doi.org/10.1016/j.neuroimage.2012.11.030 23201363 10.1016/j.neuroimage.2012.11.030 PMC3590451

[brb3887-bib-0006] Bradley, M. M. , & Lang, P. J. (1994). Measuring emotion: The self‐assessment manikin and the semantic differential. Journal of Behavior Therapy and Experimental Psychiatry, 25, 49–59. https://doi.org/10.1016/0005-7916(94)90063-9 7962581 10.1016/0005-7916(94)90063-9

[brb3887-bib-0007] Cowdrey, F. A. , Park, R. J. , Harmer, C. J. , & McCabe, C. (2011). Increased neural processing of rewarding and aversive food stimuli in recovered anorexia nervosa. Biological Psychiatry, 70, 736–743. https://doi.org/10.1016/j.biopsych.2011.05.028 21714958 10.1016/j.biopsych.2011.05.028

[brb3887-bib-0008] Delorme, A. , & Makeig, S. (2004). EEGLAB: An open source toolbox for analysis of single‐trial EEG dynamics including independent component analysis. Journal of Neuroscience Methods, 134, 9–21. https://doi.org/10.1016/j.jneumeth.2003.10.009 15102499 10.1016/j.jneumeth.2003.10.009

[brb3887-bib-0009] Diana, R. A. , Yonelinas, A. P. , & Ranganath, C. (2007). Imaging recollection and familiarity in the medial temporal lobe: A three‐component model. Trends in Cognitive Sciences, 11, 379–386. https://doi.org/10.1016/j.tics.2007.08.001 17707683 10.1016/j.tics.2007.08.001

[brb3887-bib-0010] Dorfel, D. , Werner, A. , Schaefer, M. , von Kummer, R. , & Karl, A. (2009). Distinct brain networks in recognition memory share a defined region in the precuneus. European Journal of Neuroscience, 30, 1947–1959. https://doi.org/10.1111/j.1460-9568.2009.06973.x 19895564 10.1111/j.1460-9568.2009.06973.x

[brb3887-bib-0011] Duzel, E. , Habib, R. , Schott, B. , Schoenfeld, A. , Lobaugh, N. , McIntosh, A. R. , … Heinze, H. J. (2003). A multivariate, spatiotemporal analysis of electromagnetic time‐frequency data of recognition memory. NeuroImage, 18, 185–197. https://doi.org/10.1016/S1053-8119(02)00031-9 12595175 10.1016/s1053-8119(02)00031-9

[brb3887-bib-0012] Evero, N. , Hackett, L. C. , Clark, R. D. , Phelan, S. , & Hagobian, T. A. (2012). Aerobic exercise reduces neuronal responses in food reward brain regions. Journal of Applied Physiology, 112, 1612–1619. https://doi.org/10.1152/japplphysiol.01365.2011 22383502 10.1152/japplphysiol.01365.2011

[brb3887-bib-0013] Fichter, M. M. , Herpertz, S. , Quadflieg, N. , & Herpertz‐Dahlmann, B. (1998). Structured Interview for anorexic and bulimic disorders for DSM‐IV and ICD‐10: Updated (third) revision. International Journal of Eating Disorders, 24, 227–249. https://doi.org/10.1002/(ISSN)1098-108X 9741034 10.1002/(sici)1098-108x(199811)24:3<227::aid-eat1>3.0.co;2-o

[brb3887-bib-0014] Garcia‐Garcia, I. , Narberhaus, A. , Marques‐Iturria, I. , Garolera, M. , Radoi, A. , Segura, B. , … Jurado, M. A. (2013). Neural responses to visual food cues: Insights from functional magnetic resonance imaging. European Eating Disorders Review, 21, 89–98. https://doi.org/10.1002/erv.2216 23348964 10.1002/erv.2216

[brb3887-bib-0015] Godier, L. R. , Scaife, J. C. , Braeutigam, S. , & Park, R. J. (2016). Enhanced early neuronal processing of food pictures in anorexia nervosa: A magnetoencephalography study. Psychiatry Journal, 2016, 1795901. 27525258 10.1155/2016/1795901 PMC4976260

[brb3887-bib-0016] Goldstone, A. P. , Prechtl de Hernandez, C. G. , Beaver, J. D. , Muhammed, K. , Croese, C. , Bell, G. , … Bell, J. D. (2009). Fasting biases brain reward systems towards high‐calorie foods. European Journal of Neuroscience, 30, 1625–1635. https://doi.org/10.1111/j.1460-9568.2009.06949.x 19811532 10.1111/j.1460-9568.2009.06949.x

[brb3887-bib-0017] Haber, S. N. , & Knutson, B. (2010). The reward circuit: Linking primate anatomy and human imaging. Neuropsychopharmacology, 35, 4–26. https://doi.org/10.1038/npp.2009.129 19812543 10.1038/npp.2009.129 PMC3055449

[brb3887-bib-0018] Hampshire, A. , Chamberlain, S. R. , Monti, M. M. , Duncan, J. , & Owen, A. M. (2010). The role of the right inferior frontal gyrus: Inhibition and attentional control. NeuroImage, 50, 1313–1319. https://doi.org/10.1016/j.neuroimage.2009.12.109 20056157 10.1016/j.neuroimage.2009.12.109 PMC2845804

[brb3887-bib-0019] Harsay, H. A. , Spaan, M. , Wijnen, J. G. , & Ridderinkhof, K. R. (2012). Error awareness and salience processing in the oddball task: Shared neural mechanisms. Frontiers in Human Neuroscience, 6, 246. 22969714 10.3389/fnhum.2012.00246 PMC3427876

[brb3887-bib-0020] Hauk, O. , Davis, M. H. , Ford, M. , Pulvermuller, F. , & Marslen‐Wilson, W. D. (2006). The time course of visual word recognition as revealed by linear regression analysis of ERP data. NeuroImage, 30, 1383–1400. https://doi.org/10.1016/j.neuroimage.2005.11.048 16460964 10.1016/j.neuroimage.2005.11.048

[brb3887-bib-0021] Hermans, D. , Pieters, G. , & Eelen, P. (1998). Implicit and explicit memory for shape, body weight, and food‐related words in patients with anorexia nervosa and nondieting controls. Journal of Abnormal Psychology, 107, 193–202. https://doi.org/10.1037/0021-843X.107.2.193 9604549 10.1037//0021-843x.107.2.193

[brb3887-bib-0022] Herzmann, G. , Jin, M. , Cordes, D. , & Curran, T. (2012). A within‐subject ERP and fMRI investigation of orientation‐specific recognition memory for pictures. Cognitive Neuroscience, 3, 174–192. https://doi.org/10.1080/17588928.2012.669364 22984367 10.1080/17588928.2012.669364 PMC3439853

[brb3887-bib-0023] Hoppstadter, M. , Baeuchl, C. , Diener, C. , Flor, H. , & Meyer, P. (2015). Simultaneous EEG‐fMRI reveals brain networks underlying recognition memory ERP old/new effects. NeuroImage, 116, 112–122. https://doi.org/10.1016/j.neuroimage.2015.05.026 25988228 10.1016/j.neuroimage.2015.05.026

[brb3887-bib-0024] Jacoby, L. L. (1991). A process dissociation framework: Separating automatic from intentional use of memory. Journal of Memory and Language, 30, 513–541. https://doi.org/10.1016/0749-596X(91)90025-F

[brb3887-bib-0025] Japee, S. , Holiday, K. , Satyshur, M. D. , Mukai, I. , & Ungerleider, L. G. (2015). A role of right middle frontal gyrus in reorienting of attention: A case study. Frontiers in Systems Neuroscience, 9, 23. 25784862 10.3389/fnsys.2015.00023 PMC4347607

[brb3887-bib-0026] Karhunen, L. J. , Vanninen, E. J. , Kuikka, J. T. , Lappalainen, R. I. , Tiihonen, J. , & Uusitupa, M. I. (2000). Regional cerebral blood flow during exposure to food in obese binge eating women. Psychiatry Research, 99, 29–42. https://doi.org/10.1016/S0925-4927(00)00053-6 10891647 10.1016/s0925-4927(00)00053-6

[brb3887-bib-0027] Kaufmann, C. , Beucke, J. C. , Preusse, F. , Endrass, T. , Schlagenhauf, F. , Heinz, A. , … Kathmann, N. (2013). Medial prefrontal brain activation to anticipated reward and loss in obsessive‐compulsive disorder. NeuroImage Clinical, 2, 212–220. https://doi.org/10.1016/j.nicl.2013.01.005 24179774 10.1016/j.nicl.2013.01.005 PMC3777673

[brb3887-bib-0028] Kayser, J. , Tenke, C. E. , Kroppmann, C. J. , Fekri, S. , Alschuler, D. M. , Gates, N. A. , … Bruder, G. E. (2010). Current source density (CSD) old/new effects during recognition memory for words and faces in schizophrenia and in healthy adults. International Journal of Psychophysiology, 75, 194–210. https://doi.org/10.1016/j.ijpsycho.2009.12.001 19995583 10.1016/j.ijpsycho.2009.12.001 PMC2856653

[brb3887-bib-0029] Koshino, H. , Minamoto, T. , Ikeda, T. , Osaka, M. , Otsuka, Y. , & Osaka, N. (2011). Anterior medial prefrontal cortex exhibits activation during task preparation but deactivation during task execution. PLoS ONE, 6, e22909 https://doi.org/10.1371/journal.pone.0022909 21829668 10.1371/journal.pone.0022909 PMC3148238

[brb3887-bib-0030] Kringelbach, M. L. (2004). Food for thought: Hedonic experience beyond homeostasis in the human brain. Neuroscience, 126, 807–819. https://doi.org/10.1016/j.neuroscience.2004.04.035 15207316 10.1016/j.neuroscience.2004.04.035

[brb3887-bib-0031] Leland, D. S. , & Pineda, J. A. (2006). Effects of food‐related stimuli on visual spatial attention in fasting and nonfasting normal subjects: Behavior and electrophysiology. Clinical Neurophysiology, 117, 67–84. https://doi.org/10.1016/j.clinph.2005.09.004 16337433 10.1016/j.clinph.2005.09.004

[brb3887-bib-0032] Mandler, G. (1980). Recognizing: The judgment of previous occurence. Psychological Review, 87, 252–271. https://doi.org/10.1037/0033-295X.87.3.252

[brb3887-bib-0033] Maratos, E. J. , Allan, K. , & Rugg, M. D. (2000). Recognition memory for emotionally negative and neutral words: An ERP study. Neuropsychologia, 38, 1452–1465. https://doi.org/10.1016/S0028-3932(00)00061-0 10906371 10.1016/s0028-3932(00)00061-0

[brb3887-bib-0034] Mecklinger, A. (2000). Interfacing mind and brain: A neurocognitive model of recognition memory. Psychophysiology, 37, 565–582. https://doi.org/10.1111/1469-8986.3750565 11037034

[brb3887-bib-0035] Mehta, J. , Jerger, S. , Jerger, J. , & Martin, J. (2009). Electrophysiological correlates of word comprehension: Event‐related potential (ERP) and independent component analysis (ICA). International Journal of Audiology, 48, 1–11. https://doi.org/10.1080/14992020802527258 19173108 10.1080/14992020802527258

[brb3887-bib-0036] Nelson, D. L. , Kitto, K. , Galea, D. , McEvoy, C. L. , & Bruza, P. D. (2013). How activation, entanglement, and searching a semantic network contribute to event memory. Memory & Cognition, 41, 797–819. https://doi.org/10.3758/s13421-013-0312-y 23645391 10.3758/s13421-013-0312-y

[brb3887-bib-0037] Nijs, I. M. , Franken, I. H. , & Muris, P. (2010). Food‐related Stroop interference in obese and normal‐weight individuals: Behavioral and electrophysiological indices. Eating Behaviors, 11, 258–265. https://doi.org/10.1016/j.eatbeh.2010.07.002 20850061 10.1016/j.eatbeh.2010.07.002

[brb3887-bib-0038] Nikendei, C. , Friederich, H. C. , Weisbrod, M. , Walther, S. , Sharma, A. , Herzog, W. , … Bender, S. (2012). Event‐related potentials during recognition of semantic and pictorial food stimuli in patients with anorexia nervosa and healthy controls with varying internal states of hunger. Psychosomatic Medicine, 74, 136–145. https://doi.org/10.1097/PSY.0b013e318242496a 22291203 10.1097/PSY.0b013e318242496a

[brb3887-bib-0039] Nikendei, C. , Funiok, C. , Pfuller, U. , Zastrow, A. , Aschenbrenner, S. , Weisbrod, M. , … Friederich, H. C. (2011). Memory performance in acute and weight‐restored anorexia nervosa patients. Psychological Medicine, 41, 829–838. https://doi.org/10.1017/S0033291710001121 20529417 10.1017/S0033291710001121

[brb3887-bib-0040] Nikendei, C. , Schild, S. , Voelkl, M. , Herzog, W. , & Zipfel, S. (2005). Anorexia nervosa: Self‐assessment of eating disorder‐related words and pictorial stimuli. Psychotherapie Psychosomatik Medizinische Psychologie, 55, 93.

[brb3887-bib-0041] Nikendei, C. , Weisbrod, M. , Schild, S. , Bender, S. , Walther, S. , Herzog, W. , … Friederich, H. C. (2008). Anorexia nervosa: Selective processing of food‐related word and pictorial stimuli in recognition and free recall tests. International Journal of Eating Disorders, 41, 439–447. https://doi.org/10.1002/(ISSN)1098-108X 18348282 10.1002/eat.20518

[brb3887-bib-0042] Pietrowsky, R. , Krug, R. , Fehm, H. L. , & Born, J. (2002). Food deprivation fails to affect preoccupation with thoughts of food in anorectic patients. British Journal of Clinical Psychology, 41, 321–326. https://doi.org/10.1348/014466502760379172 12396259 10.1348/014466502760379172

[brb3887-bib-0043] Piqueras‐Fiszman, B. , Kraus, A. A. , & Spence, C. (2014). “Yummy” versus “Yucky”! Explicit and implicit approach‐avoidance motivations towards appealing and disgusting foods. Appetite, 78, 193–202. https://doi.org/10.1016/j.appet.2014.03.029 24709484 10.1016/j.appet.2014.03.029

[brb3887-bib-0044] Ptak, R. (2012). The frontoparietal attention network of the human brain: Action, saliency, and a priority map of the environment. Neuroscientist, 18, 502–515. https://doi.org/10.1177/1073858411409051 21636849 10.1177/1073858411409051

[brb3887-bib-0045] Rugg, M. D. , & Curran, T. (2007). Event‐related potentials and recognition memory. Trends in Cognitive Sciences, 11, 251–257. https://doi.org/10.1016/j.tics.2007.04.004 17481940 10.1016/j.tics.2007.04.004

[brb3887-bib-0046] Schloerscheidt, A. M. , & Rugg, M. D. (2004). The impact of change in stimulus format on the electrophysiological indices of recognition. Neuropsychologia, 42, 451–466. https://doi.org/10.1016/j.neuropsychologia.2003.08.010 14728919 10.1016/j.neuropsychologia.2003.08.010

[brb3887-bib-0047] Skinner, E. I. , & Fernandes, M. A. (2007). Neural correlates of recollection and familiarity: A review of neuroimaging and patient data. Neuropsychologia, 45, 2163–2179. https://doi.org/10.1016/j.neuropsychologia.2007.03.007 17445844 10.1016/j.neuropsychologia.2007.03.007

[brb3887-bib-0048] Spaniol, J. , Davidson, P. S. , Kim, A. S. , Han, H. , Moscovitch, M. , & Grady, C. L. (2009). Event‐related fMRI studies of episodic encoding and retrieval: Meta‐analyses using activation likelihood estimation. Neuropsychologia, 47, 1765–1779. https://doi.org/10.1016/j.neuropsychologia.2009.02.028 19428409 10.1016/j.neuropsychologia.2009.02.028

[brb3887-bib-0049] Stockburger, J. , Schmalzle, R. , Flaisch, T. , Bublatzky, F. , & Schupp, H. T. (2009). The impact of hunger on food cue processing: An event‐related brain potential study. NeuroImage, 47, 1819–1829. https://doi.org/10.1016/j.neuroimage.2009.04.071 19409497 10.1016/j.neuroimage.2009.04.071

[brb3887-bib-0050] Summerfield, C. , & Mangels, J. A. (2005). Coherent theta‐band EEG activity predicts item‐context binding during encoding. NeuroImage, 24, 692–703. https://doi.org/10.1016/j.neuroimage.2004.09.012 15652304 10.1016/j.neuroimage.2004.09.012

[brb3887-bib-0051] Suslow, T. (2009). Estimating verbal intelligence in unipolar depression: Comparison of word definition and word recognition. Nordic Journal of Psychiatry, 63, 120–123. https://doi.org/10.1080/08039480802316010 18985516 10.1080/08039480802316010

[brb3887-bib-0052] Talati, A. , & Hirsch, J. (2005). Functional specialization within the medial frontal gyrus for perceptual go/no‐go decisions based on “what”, “when”, and “where” related information: An fMRI study. Journal of Cognitive Neuroscience, 17, 981–993. https://doi.org/10.1162/0898929054475226 16102231 10.1162/0898929054475226

[brb3887-bib-0053] Tataranni, P. A. , & DelParigi, A. (2003). Functional neuroimaging: A new generation of human brain studies in obesity research. Obesity Reviews, 4, 229–238. https://doi.org/10.1046/j.1467-789X.2003.00111.x 14649373 10.1046/j.1467-789x.2003.00111.x

[brb3887-bib-0054] Terhoeven, V. , Kallen, U. , Ingenerf, K. , Aschenbrenner, S. , Weisbrod, M. , Herzog, W. , Brockmeyer, T. , Friederich, H. C. , & Nikendei, C. (2016). Meaningful memory in acute anorexia nervosa patients‐comparing recall, learning, and recognition of semantically related and semantically unrelated word stimuli. European Eating Disorders Review, 25, 89–97. 28032373 10.1002/erv.2496

[brb3887-bib-0055] van Tol, M. J. , Demenescu, L. R. , van der Wee, N. J. , Kortekaas, R. , Marjan, M. A. N. , Boer, J. A. , … Veltman, D. J. (2012). Functional magnetic resonance imaging correlates of emotional word encoding and recognition in depression and anxiety disorders. Biological Psychiatry, 71, 593–602. 22206877 10.1016/j.biopsych.2011.11.016

[brb3887-bib-0056] Veenstra, E. M. , & de Jong, P. J. (2010). Restrained eaters show enhanced automatic approach tendencies towards food. Appetite, 55, 30–36. https://doi.org/10.1016/j.appet.2010.03.007 20298730 10.1016/j.appet.2010.03.007

[brb3887-bib-0057] Vilberg, K. L. , & Rugg, M. D. (2008). Memory retrieval and the parietal cortex: A review of evidence from a dual‐process perspective. Neuropsychologia, 46, 1787–1799. https://doi.org/10.1016/j.neuropsychologia.2008.01.004 18343462 10.1016/j.neuropsychologia.2008.01.004 PMC2488316

[brb3887-bib-0058] Wittchen, H. U. , Zaudig, M. , & Fydrich, T. (1997). Strukturiertes klinisches interview für DSM‐IV (SKID‐I und SKID‐II). Göttingen, Germany: Hogrefe.

[brb3887-bib-0059] Yonelinas, A. P. , Otten, L. J. , Shaw, K. N. , & Rugg, M. D. (2005). Separating the brain regions involved in recollection and familiarity in recognition memory. Journal of Neuroscience, 25, 3002–3008. https://doi.org/10.1523/JNEUROSCI.5295-04.2005 15772360 10.1523/JNEUROSCI.5295-04.2005 PMC6725129

[brb3887-bib-0060] Zakzanis, K. K. , Campbell, Z. , & Polsinelli, A. (2010). Quantitative evidence for distinct cognitive impairment in anorexia nervosa and bulimia nervosa. Journal of Neuropsychology, 4, 89–106. https://doi.org/10.1348/174866409X459674 19619407 10.1348/174866409X459674

